# RetroTector online, a rational tool for analysis of retroviral elements in small and medium size vertebrate genomic sequences

**DOI:** 10.1186/1471-2105-10-S6-S4

**Published:** 2009-06-16

**Authors:** Göran Sperber, Anders Lövgren, Nils-Einar Eriksson, Farid Benachenhou, Jonas Blomberg

**Affiliations:** 1 Physiology unit, Dpt of Neuroscience, Box 593, Uppsala, Sweden; 2 Linnaeus Centre for Bioinformatics, Biomedical Centre, Box 598, 751 24 Uppsala, Sweden; 3 Section of Virology, Department of Medical Sciences, Uppsala University, Academic Hospital, 751 85 Uppsala, Sweden

## Abstract

**Background:**

The rapid accumulation of genomic information in databases necessitates rapid and specific algorithms for extracting biologically meaningful information. More or less complete retroviral sequences, also called proviral or endogenous retroviral sequences; ERVs, constitutes at least 5% of vertebrate genomes. After infecting the host, these retroviruses have integrated in germ line cells, and have then been carried in genomes for at least several 100 million years. A better understanding of structure and function of these sequences can have profound biological and medical consequences.

**Methods:**

RetroTector^© ^(ReTe) is a platform-independent Java program for identification and characterization of proviral sequences in vertebrate genomes. The full ReTe requires a local installation with a MySQL database. Although not overly complicated, the installation may take some time. A "light" version of ReTe, (RetroTector online; ROL) which does not require specific installation procedures is provided, via the World Wide Web.

**Results:**

ROL  was implemented under the Batchelor web interface (A Lövgren et al). It allows both GenBank accession number, file and FASTA cut-and-paste admission of sequences (5 to 10 000 kilobases). Up to ten submissions can be done simultaneously, allowing batch analysis of <= 100 Megabases. Jobs are shown in an IP-number specific list. Results are text files, and can be viewed with the program, RetroTectorViewer.jar (at the same site), which has the full graphical capabilities of the basic ReTe program. A detailed analysis of any retroviral sequences found in the submitted sequence is graphically presented, exportable in standard formats. With the current server, a complete analysis of a 1 Megabase sequence is complete in 10 minutes. It is possible to mask nonretroviral repetitive sequences in the submitted sequence, using host genome specific "brooms", which increase specificity.

**Discussion:**

Proviral sequences can be hard to recognize, especially if the integration occurred many million years ago. Precise delineation of LTR, *gag*, *pro*, *pol *and *env *can be difficult, requiring manual work. ROL is a way of simplifying these tasks.

**Conclusion:**

ROL provides 1. annotation and presentation of known retroviral sequences, 2. detection of proviral chains in unknown genomic sequences, with up to 100 Mbase per submission.

## Background

Genomic databases are growing dramatically. Not only are reference genomes from many organisms sequenced, the probable coming era of "personal genomics" will lead to further substantial increases. The demand for rapid and easily accessible pattern recognition tools increases concomitantly. More or less complete retroviral sequences make up a substantial part of vertebrate genomes. After infecting the host, these retroviruses have integrated in germ line cells, and have then been carried in progeny genomes for long times. Recently integrated retroviruses may still produce viral particles and could be associated with diseases. However, most ERVs are mutated to viral inactivity. Some are beneficial for the host where for example they can provide promoter elements or proteins (for a review, see [1]) Because of mutations retroviral sequences can be difficult to detect, especially the older elements which can lack recognisable retroviral protein. A better understanding of the structure and function of these sequences can have profound biological and medical consequences.

## Methods

The full version [2] of ReTe requires a local installation with a MySQL database. The basic principle of ReTe is "fragment threading". A collection of conserved motifs (mostly from proteins) are tested against the sequence to be evaluated. A chaining algorithm stitches together motif hits, which conform to limits specified in an intermotif distance table, to a "retroviral chain" which best fits the retroviral genus, distances and degree of motif similarity. Each chain is given a "retroviral score" which in essence describes the degree of conformity to a retroviral structure model. Protein translations ("puteins") are interpreted with the help of several algorithms, one being the degree of fit to an alignment of reference retroviral proteins, others focused on finding the starts and ends of the protein. In problematic cases, alternative chains and alternative putein interpretations are given. Unlike most other retrovirus-finding algorithms, ReTe is not dependent on repetition, neither of LTRs nor of proviruses. It is therefore especially important for finding low copy number proviruses, like ERVFc1 [3].

The ReTe installation may take some time for unexperienced users. With the latter in mind, we have now created a "light" version of ReTe, ROL. ROL uses the same collection of conserved motifs and the same intermotif distance table as ReTe. The results of ROL should be the same as with ReTe. However, ReTe contains several additional features, like LTR divergence, nucleotide frequency and most similar reference sequences, which are not present in ROL.

ROL does not require specific installation procedures. It can be accessed via the World Wide Web. ROL was implemented under the Batchelor web interface (A. Lövgren et al, unpublished). Batchelor is written in PHP and designed to act as a wrapper around an existing standalone application and to integrate it with the web. As a batch queue manager, Batchelor allows jobs with long run times to be submitted to the web server and scheduled for later execution by its batch queue. Users (submitters) can monitor the state of their submitted jobs (pending, running or finished) and later download the result from the queue view. The user interface is customizable through templates and extendable through user defined callback functions. Among its features are job control, statistics and (in latest releases) web service interfaces. ROL and Batchelor run on a Dell PowerEdge 2950 server with 2 × Quad Core Xeon 2.50 GHz processors, 8 GB RAM with a 64-bit Gentoo Linux operative system.

## Results

### The RetroTector online implementation

**Figure 1:**
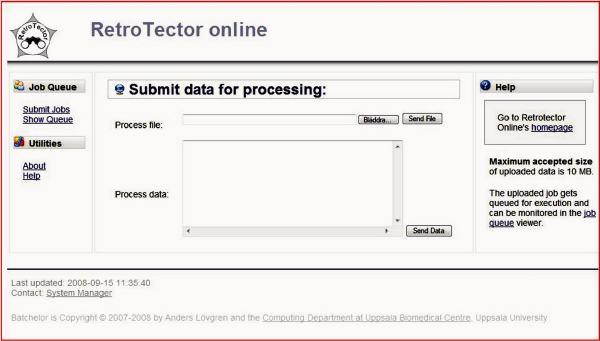
**User interface of RetroTector online**. FASTA formatted sequences, each up to 10 Mbases long, can be entered as file, by cut-and-paste, and by GenBank accession number. Up to ten sequences can be entered at the same time.

The ROL implementation , under the Batchelor web interface, allows both GenBank accession number, file and FASTA cut-and-paste admission of sequences (5 to 1000 000 kilobases) (Figure 1). Up to ten submissions can be done simultaneously. Thus, a batch analysis of <= 100 Megabases can be specified in one instance. Jobs are shown in an IP-number specific list. Results are downloadable as text files. The text files derive from 100 000 base chunks. Names of chunks which contain one or several retroviral chains detected by ReTe are shown in bold face. Each retrovirus-positive chunk can be inspected by means of the stand-alone program, RetroTectorViewer.jar, which has the full graphical capabilities of the basic ReTe program. It can export in EPS, PDF and JPG formats. Thus, a detailed analysis of any retroviral sequences found in the submitted sequence is graphically presented. With the current server, a complete analysis of a 1 Megabase sequence is complete in under 10 minutes. It is possible to mask nonretroviral repetitive sequences in the submitted sequence before analysis, using host genome specific "brooms". This increases the specificity of the analysis.

**Figure 2:**
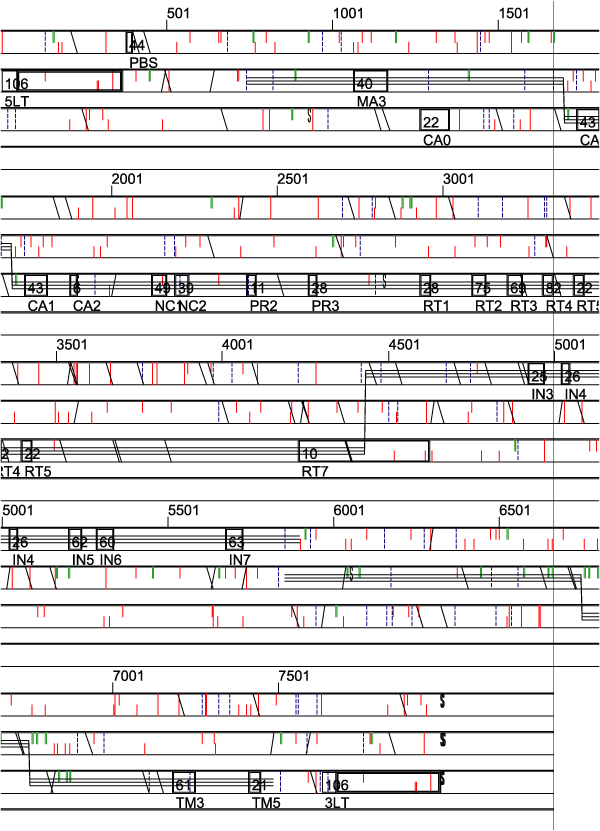
**Result from a run of the chimpanzee genome (panTro2)**. The chimpanzee version of the human HERVFc1 retroviral sequence [3] is shown. The components LTR (5 LT & 3 LT), primer binding site (PBS), *gag *(internal structural proteins; motifs named CA and NC), *pro *(protease, motifs named PR), *pol *(pol gene, motifs named RT, RH and IN), *env *(envelope gene, motifs named SU and TM), and polypurine tract (PPT) are shown. Figures above each motif denote its score (0–100). The following features are also predicted: Red bars denote stop codons, blue bars start codons. Triple lines denote putative protein encoding sequences. Green bars denote putative asparagine glycosylation sites, "/" splice donor, "\ " splice acceptor, "S" "Slippery" sequence (possible frameshift sites), and "8" pseudoknot sequences (which are also possible frameshift sites).

An example (Figure 2) is the analysis of ERVFc1, a gammaretroviruslike sequence which has several ORFs and near-ORFs [1,3]. This low copy number sequence was present in previous assemblies of the human genome, but has been edited out of the hg18 assembly. The provirus shown is from the panTro2 chimpanzee genome assembly. This illustrates the importance of independent retrovirus sequence detection systems, like ReTe and ROL.

## Discussion

Several pattern recognition algorithms for recognition of retroviral sequences exist ([4-9]; for a review, see [1]). The algorithms generally have a specific purpose. Either they emphasize LTRs, repetitiveness, conserved motifs, or open reading frames. A combined search therefore may be called for. ReTe is unique in its detailed analysis of the internal proviral sequences and classification of the provirus. It is modular, which allows users to add their own program modules. Despite the existence of several programs for detection of retroviral sequences, a number of problems remain. Several portions, especially the long terminal repeats (LTRs), of retroviral genomes do not encode proteins. The detection of noncoding nucleic acid sequences is a specific instance of the more general problem of pattern recognition. The methods used in ReTe for LTR detection are mainly artificial neural networks and heuristic motif combinations [10]. These are combined with detection of conserved protein coding motifs and reading frames using pairwise and multiple amino-acid alignments. ReTe is mainly targeted to higher vertebrate genomes. For analysis of other genomes, a further optimization for detection of *Meta- *and *Pseudoviridae *sequences (also known as *gypsy *and *copia*-like sequences, respectively) is necessary. These retrovirus-related sequences are abundant in lower vertebrates, insects and plants, among others. ROL is intended for analysis of 5–10 000 Kbases of genomic sequence. For example, a batch job of 10 different cosmid sequences of 1–2 Mbases can be specified via a list of their GenBank accession numbers. Sequences of entire small chromosomes, like human chromosomes 20, 21 and 22 can be entered via cut-and-paste, accession number or as a downloaded file.

The sensitivity and specificity of ROL is the same as that of ReTe. However, a judgement regarding the choice of "brooms" must be made. Brooms are a set of preselected nonretroviral repetitive sequences prevalent in the host genome under study. Currently, brooms for higher primate, artiodactyl, rodent, bird and marsupial genomes are available at the ROL site. The brooms remove many of the weak false signals from LTR-like sequences. LTRs are difficult to detect because they usually do not code for proteins and therefore contain very few conserved motifs detectable by the unaided eye. In addition some parts of the LTRs consist of repetitive elements like C-rich or GT-rich regions that can easily be matched by other repetitive elements like ALUs. This can spur incorrect chain formation by ReTe and can result in poor detection specificities. The user may experiment with different brooms to obtain an optimal result.

## Conclusion

ROL offers a convenient way to quickly search for, annotate and present retroviral sequences in genomic sequences.

## Competing interests

The authors declare that they have no competing interests.

## Authors' contributions

GS wrote the ReTe and RetroTectorviewer Java code, and oversaw the implementation of ROL. AL wrote the Batchelor web presentation and batch processor, and adapted it to create ROL. NEE participated in the design and conception of ROL. FB participated in the writing of the manuscript. JB is the retrovirologist behind ReTe, tested the user interface, provided ideas on useful features and participated in the writing of the manuscript.

## References

[B1] Blikstad V, Benachenhou F, Sperber GO, Blomberg J (2008). Evolution of human endogenous retroviral sequences: a conceptual account. Cell Mol Life Sci.

[B2] Sperber GO, Airola T, Jern P, Blomberg J (2007). Automated recognition of retroviral sequences in genomic data – RetroTector. Nucleic acids research.

[B3] Benit L, Calteau A, Heidmann T (2003). Characterization of the low-copy HERV-Fc family: evidence for recent integrations in primates of elements with coding envelope genes. Virology.

[B4] McCarthy EM, McDonald JF (2003). LTR_STRUC: a novel search and identification program for LTR retrotransposons. Bioinformatics.

[B5] Villesen P, Aagaard L, Wiuf C, Pedersen FS (2004). Identification of endogenous retroviral reading frames in the human genome. Retrovirology.

[B6] Jurka J, Klonowski P, Dagman V, Pelton P (1996). CENSOR – a program for identification and elimination of repetitive elements from DNA sequences. Comput Chem.

[B7] Jurka J, Kapitonov VV, Pavlicek A, Klonowski P, Kohany O, Walichiewicz J (2005). Repbase Update, a database of eukaryotic repetitive elements. Cytogenet Genome Res.

[B8] de Parseval N, Lazar V, Casella JF, Benit L, Heidmann T (2003). Survey of human genes of retroviral origin: identification and transcriptome of the genes with coding capacity for complete envelope proteins. Journal of virology.

[B9] Smit AF (1999). Interspersed repeats and other mementos of transposable elements in mammalian genomes. Curr Opin Genet Dev.

[B10] Durbin R, Eddy S, Krogh A, Mitchison G (2005). Biological Sequence analysis.

